# Dataset of distribution, habitats and conservation status of the Indian pangolin (*Manis crassicaudata*) in Sri Lanka

**DOI:** 10.1016/j.dib.2019.104999

**Published:** 2019-12-16

**Authors:** Hasitha Karawita, Priyan Perera

**Affiliations:** 1 Department of Forestry and Environmental Science, University of Sri Jayewardenepura, Sri Lanka; 2 IUCN SSC Pangolin Specialist Group, C/o Zoological Society of London, Regent's Park, London, NW1 4RY, United Kingdom

**Keywords:** Indian pangolin, Distribution, Habitats, Crime, Rescue

## Abstract

Indian pangolin (*Manis crassicaudata*) is the only pangolin species present in Sri Lanka. There is no comprehensive assessment of its ecology or conservation status carried out in the Sri Lankan context. The dataset described herein is a compilation of information on the distribution, habitats and conservation status of Indian pangolins in Sri Lanka which is collected from a variety of primary and secondary data sources. All information included in the dataset has been recorded between January 2000 and December 2018. The data on distribution, crimes and rescue activities involving Indian pangolins all over the country were collected from the registries maintained by the Department of Wildlife Conservation, Department of National Zoological Gardens and non governmental organizations committed to the conservation of wildlife in Sri Lanka. Verified records from mass media and reliable field data gathered by the authors and their contact networks were further included in the dataset. The data on the distribution can be analyzed to identify the different habitats of the Indian pangolins and their abundance in different climatic zones. The data on distribution include the recorded area, habitat and approximate GPS coordinates of the recorded locality. The data on crimes involving pangolins was extracted from the offices of the Department of Wildlife Conservation which record the crime, date of crime, approximate GPS coordinates of localities where crimes occurred, nature of the crime and fines/actions taken against the offenders. Data on the rescue events include approximate GPS coordinates of the places where the Indian pangolins were rescued, health conditions at the point of rescue and post rescue status [1].

**Table:** Specifications Table

Subject	Ecology
Specific subject area	Ecology and identification of threats
Type of data	Tables
How data were acquired	The data on distribution, crimes and rescue activities involving pangolins all over the country were collected from the registries maintained by the Department of Wildlife Conservation, Department of National Zoological Gardens, Sri Lanka and nongovernmental organizations committed to the conservation of wildlife. Records from mass media and field data gathered by the authors and their contact networks were further included to the data set after verifying the authenticity.
Data format	Raw and Filtered
Parameters for data collection	Due to the difficulty of retrieving old records, only the cases reported between January 2000 to December 2018 were collected.
Description of data collection	The records on crimes and rescue events involving Indian pangolins were collected from the registries maintained by the beat offices, range offices, regional offices and national park offices of the Department of Wildlife Conservation (DWC), Sri Lanka. Furthermore, records on rescued Indian pangolins were collected from animal rescue records maintained at the Pinnawala Zoological Garden of the Department of National Zoological Gardens, Sri Lanka, animal rescue centers managed by the DWC, and similar facilities operated by non-governmental organizations. The records on distribution is produced by combining the rescue and crime data collected from the above sources, and verified media (newspapers and web-media reports) records.
Data source location	**Institution:** Department of Wildlife Conservation Department of National Zoological Gardens Wildlife health care center managed by NGO **City/Town/Region**: Department of Wildlife Conservation – beet/range/national park/regional offices and health care centers in island wide, Sri Lanka Department of National Zoological Gardens –National Zoological Garden, Pinnawala, Sri Lanka Wild life health care center managed by NGO –Hiyare, Sri Lanka **Country: Sri Lanka** 5° 55′ to 9° 51′ North latitude and between 79° 42′ to 81° 53′ East longitude
Data accessibility	Date is with this Article
Related research article	Author's name: Priyan Perera and Hasitha.Karawita Title: An Update of Distribution, Habitats and Conservation Status of the Indian Pangolin (*Manis crassicaudata*) in Sri Lanka Journal: Global Ecology and Conservation DOI: https://doi.org/10.1016/j.gecco.2019.e00799

**Table:** 

**Value of the Data** • The dataset in this article includes updated information on distribution, and habitats of the Indian Pangolin present in Sri Lanka • The dataset holds national and global importance in assessing the conservation status of the Indian Pangolin (*Manis crassicaudata*). • The records on crimes involving pangolins will be useful to understand the nature and trends in illegal trading of pangolins and their derivatives in local and international markets. • Data on rescues and crimes related to Indian Pangolin has implications on the conservation planning of this species in Sri Lanka

## 1 Data description

The dataset described in this article has been used to update the distribution, habitats and conservation status of the Indian Pangolin (*Manis crassicaudata*) in Sri Lanka [1]. Data on the distribution of Indian pangolin include recorded areas, habitats of the species and approximate GPS coordinates of recorded areas throughout Sri Lanka (Table 1). The recorded habitats were further classified according to the different habitat types as described in the IUCN, habitats classification scheme 2019 (version 3.1). Data on the crimes involving the Indian pangolins consist the nature of crime, date of crime, fines charged from the offenders or the present conditions of the investigations, recorded locality, approximate GPS coordinates of those localities and the DWC office which reports the particular crime related to the Indian pangolin (Table 2). The nature of crime describes the category of crime which may include the killing of pangolin, possession or selling flesh, possession of scales and other body parts or nonpurposive kills due to human activities. Data on the rescue records related to the Indian Pangolins include the locality where the rescue event was carried; approximate GPS coordinates of those localities, health conditions of pangolins at the event of rescue and post-rescue status (Table 3).

**Table 1:** Data on the recorded areas, habitats and approximate GPS coordinates of recorded localities of Indian Pangolins found in Sri Lanka.

Area	Habitat	Longitude	Latitude
Kanakapuram	Tropical Dry Shrubland	80°23′14.07″E	9°23′0.27″N
Kilinochchi	Tropical Dry Shrubland	80°22′37.20″E	9°22′49.04″N
Trincomalee	Tropical Dry Shrubland	81°12′54.76″E	8°35′14.51″N
Kahatagas wewa	Tropical Heavily Degraded Former Forest	80°53′25.83″E	8°26′42.41″N
Puliyankulama	Tropical Dry Shrubland	80°34′44.27″E	8°28′38.25″N
Kakerawa	Tropical Dry Forest	80°35′37.80″E	8° 2′31.57″N
Palugas wewa	Tropical Heavily Degraded Former Forest	80°41′17.42″E	8° 3′37.59″N
Mullaitivu	Tropical Dry Forest	80°48′47.3″E	9°15′50.9″N
Pubbogama	Tropical Dry Forest	80°35′48.55″E	7°55′41.09″N
Wilpattu	Tropical Dry Forest	80° 2′51.50″E	8°27′22.94″N
Hunuvila gama	Tropical Dry Shrubland	80°10′11.30″E	8°19′2.14″N
Nikaweratiya	Tropical Dry Shrubland	80° 7′54.09″E	7°44′46.99″N
Maduru oya	Tropical Dry Forest	81°11′9.67″E	7°39′24.87″N
Panamure	Tropical Moist Shrubland	80°46′6.78″E	6°20′37.77″N
Kalamatiya	Tropical Dry Shrubland	80°56′14.50″E	6° 5′31.87″N
Udawalawa	Tropical Dry Forest	80°49′26.28″E	6°25′6.61″N
Kolambage ara	Tropical Heavily Degraded Former Forest	80°46′49.17″E	6°25′6.08″N
Dikwella	Tropical Moist Shrubland	80°41′42.38″E	5°58′18.04″N
Kahawatta	Tropical Moist Lowland Forest	80°34′27.83″E	6°34′57.18″N
Lellopitiya	Tropical Moist Lowland Forest	80°28′55.09″E	6°40′0.29″N
Udawalawa	Tropical Dry Shrubland	80°48′16.65″E	6°28′20.57″N
Balangoda	Tropical Moist Shrubland	80°42′17.31″E	6°40′0.70″N
Dahaiyagala	Tropical Heavily Degraded Former Forest	80°34′13.00″E	7°31′45.99″N
Kalthota	Moist Savanna	80°52′15.55″E	6°39′33.84″N
Palabaddala	Moist Savanna	80°27′35.45″E	6°47′42.06″N
Balangoda	Tropical Moist Lowland Forest	80°40′1.45″E	6°38′59.19″N
Polpithigama	Tropical Heavily Degraded Former Forest	80°24′42.47″E	7°48′45.16″N
Kurunagala	Tropical Heavily Degraded Former Forest	80°21′43.68″E	7°32′27.05″N
Malkaduwawa	Tropical Heavily Degraded Former Forest	80°20′9.08″E	7°28′7.68″N
Giriulla	Tropical Heavily Degraded Former Forest	80° 7′23.95″E	7°19′54.37″N
Rajanganaya	Tropical Dry Shrubland	80°11′47.20″E	8° 9′56.59″N
Nikawaratiya	Tropical Dry Shrubland	80° 7′54.09″E	7°44′46.99″N
Polpithigama	Tropical Dry Shrubland	80°24′42.65″E	7°48′50.64″N
Kobeyigane	Tropical Dry Shrubland	80° 7′33.99″E	7°39′18.49″N
Yapahuwa	Tropical Heavily Degraded Former Forest	80°17′55.37″E	7°49′39.56″N
Kurunagala	Tropical Heavily Degraded Former Forest	80°21′59.41″E	7°30′58.42″N
Thiththawella	Tropical Heavily Degraded Former Forest	80°20′50.78″E	7°30′59.06″N
Sigiriya	Tropical Dry Shrubland	80°45′36.58″E	7°57′18.18″N
Katukaliyawa	Tropical Heavily Degraded Former Forest	80°35′18.31″E	8°21′8.74″N
Sigiriya	Tropical Dry Shrubland	80°45′48.04″E	7°57′27.35″N
Bandimaawa	Rural Gardens	80°47′18.33″E	8° 0′19.12″N
Diyabeduma	Tropical Heavily Degraded Former Forest	80°53′59.42″E	7°56′38.39″N
Trincomalee	Tropical Heavily Degraded Former Forest	81°10′35.75″E	8°33′19.53″N
Minneriya	Tropical Dry Shrubland	80°54′44.43″E	8° 3′9.99″N
Herathgama	Tropical Dry Forest	80°12′15.18″E	7°39′50.69″N
Pandulagama	Rural Gardens	80°21′16.45″E	8°19′40.66″N
Habarana	Tropical Dry Forest	80°44′28.70″E	8° 2′12.09″N
Minneriya	Tropical Dry Shrubland	80°54′40.01″E	8° 2′48.55″N
Deyiyandara	Tropical Moist Shrubland	80°36′07.9″E	6°09′16.3″N
Buththala	Tropical Dry Forest	81°14′52.5″E	6°39′00.6″N
Yatiyana	Tropical Moist Shrubland	80°35′54.5″E	6°01′29.6″N
Tissa	Tropical Dry Shrubland	81°16′28.0″E	6°18′52.7″N
Lunugamvehera	Tropical Dry Forest	81°12′04.3″E	6°21′10.5″N
Ranawaranawa	Rural Gardens	81°08′55.2″E	6°24′24.6″N
Wallawaya	Tropical Dry Shrubland	81°05′49.2″E	6°44′02.8″N
Okkampitiya	Plantations	81°18′37.4″E	6°45′27.9″N
Thalakolawewa	Tropical Dry Forest	81°15′51.4″E	6°43′09.3″N
Paranigama	Plantations	81°05′49.1″E	8°03′51.4″N
Kethsirigama	Tropical Dry Shrubland	81°39′36.2″E	7°12′28.1″N
Nawaththadu	Tropical Dry Shrubland	79°50′34.7″E	8°10′00.7″N
Norochcholai	Tropical Dry Shrubland	79°42′54.5″E	8°02′30.4″N
Ilanthadiya	Tropical Dry Shrubland	79°42′52.6″E	8°04′03.7″N
Ilanthadiya	Tropical Dry Shrubland	79°43′36.9″E	8°10′39.0″N
Ekkala	Tropical Dry Shrubland	79°51′01.4″E	8°08′47.9″N
Baduraliya	Tropical Moist Lowland Forest	80°14′20.4″E	6°31′02.0″N
Badugama	Tropical Moist Lowland Forest	80°07′17.1″E	6°32′25.7″N
Miriswaththa	Tropical Moist Lowland Forest	80°08′43.1″E	6°57′41.2″N
Wanduramba	Tropical Moist Lowland Forest	80°15′48.5″E	6°07′58.4″N
Unawatuna	Tropical Moist Lowland Forest	80°15′42.6″E	6°00′33.2″N
Wanduramba	Tropical Moist Lowland Forest	80°14′44.9″E	6°08′29.7″N
Baddegama	Tropical Moist Lowland Forest	80°11′31.6″E	6°10′37.5″N
Karandeniya	Tropical Moist Lowland Forest	80°06′10.2″E	6°16′26.1″N
Poddala	Tropical Moist Lowland Forest	80°12′46.5″E	6°07′27.7″N
Yakkalamulla	Tropical Moist Lowland Forest	80°21′09.6″E	6°05′30.4″N
Elpitiya	Tropical Moist Lowland Forest	80°09′13.6″E	6°17′17.8″N
Udugama	Tropical Moist Lowland Forest	80°21′02.8″E	6°14′09.1″N
Imaduwa	Tropical Moist Lowland Forest	80°23′10.0″E	6°02′05.8″N
Hunnasgiriya	Tropical Moist Montane Forest	80°41′32.1″E	7°23′18.0″N
Hanguranketha	Tropical Moist Montane Forest	80°48′33.1″E	7°10′11.9″N
Wirambugedara	Tropical Heavily Degraded Former Forest	80°14′22.7″E	7°28′35.4″N
Katupotha	Tropical Dry Shrubland	80°10′30.0″E	7°32′50.9″N
Horambawa	Tropical Dry Shrubland	80°11′11.1″E	7°28′51.4″N
Hettipola	Tropical Dry Shrubland	80°04′46.0″E	7°35′43.4″N
Narammala	Tropical Dry Shrubland	80°14′50.6″E	7°25′05.2″N
Hettipola	Tropical Dry Shrubland	80°04′45.0″E	7°35′49.9″N
Madalagama	Tropical Moist Shrubland	80°30′05.3″E	6°33′53.8″N
Wariyapola	Tropical Moist Shrubland	80°24′17.4″E	7°37′15.3″N
Kuliyapitiya	Tropical Dry Shrubland	80°03′30.1″E	7°28′32.2″N
Pallegama	Tropical Moist Shrubland	80°03′25.7″E	7°17′44.6″N
Thalageya	Tropical Dry Shrubland	80°17′34.3″E	7°28′03.3″N
Hulogedara	Tropical Dry Shrubland	80°11′19.4″E	7°47′45.3″N
Aliyawa	Tropical Dry Shrubland	80°10′01.5″E	7°45′21.9″N
Nillegama	Tropical Dry Shrubland	80°09′50.5″E	7°45′57.3″N
Kobeyigane	Tropical Dry Shrubland	80°08′00.8″E	7°39′22.7″N
Galaha	Tropical Moist Lowland Forest	80°35′43.0″E	7°16′03.1″N
Galway's Land	Tropical Moist Montane Forest	80°46′37.2″E	6°58′01.1″N
Hingurukaduwa	Moist Savanna	81°08′25.3″E	6°49′16.7″N
Bandarawela	Tropical Moist Montane Forest	80°59′30.2″E	6°49′07.8″N
Diyathalawa	Tropical Moist Montane Forest	80°57′59.2″E	6°47′57.7″N
Thelijjawila	Tropical Moist Lowland Forest	80°29′35.9″E	6°01′43.1″N
Waligama	Tropical Moist Lowland Forest	80°29′25.5″E	6°01′38.3″N
Walihinda	Tropical Heavily Degraded Former Forest	80°28′02.9″E	5°58′54.4″N
Yakkalamulla	Tropical Moist Lowland Forest	80°21′48.4″E	6°06′36.9″N
Pinnaduwa	Tropical Moist Lowland Forest	80°16′25.2″E	6°04′07.4″N
Udugama	Tropical Moist Lowland Forest	80°20′40.3″E	6°13′53.5″N
Uhana	Tropical Moist Lowland Forest	81°38′27.6″E	7°22′51.4″N
Udawalawa	Tropical Dry Shrubland	80°48′11.5″E	6°26′45.2″N
Iginiyagala	Tropical Dry Forest	81°33′05.8″E	7°12′04.4″N
Uhana	Tropical Dry Forest	81°38′09.8″E	7°22′25.9″N
Galapita gala	Tropical Dry Forest	81°39′30.7″E	7°28′02.5″N
Maduru oya	Tropical Heavily Degraded Former Forest	81°12′58.1″E	7°38′57.0″N
Ampara	Tropical Dry Shrubland	81°40′04.2″E	7°13′59.7″N
Ganewalpola	Tropical Dry Shrubland	80°37′40.50″E	8° 5′15.40″N
Ipalogama	Tropical Dry Shrubland	80°31′57.57″E	8° 4′22.72″N
Periya madu	Tropical Dry Shrubland	80°58′53.30″E	8°52′29.40″N
Madu	Tropical Dry Shrubland	80°12′15.28″E	8°51′22.90″N
Horowupathana	Tropical Dry Forest	80°50′6.04″E	8°33′33.48″N
Anuradhapura	Tropical Dry Forest	80°21′4.88″E	8°18′28.35″N
Mihinthalaya	Tropical Dry Forest	80°30′16.43″E	8°21′8.75″N
Seeppukulama	Tropical Dry Shrubland	80°35′24.90″E	8°22′50.89″N
Eluwankulama	Tropical Dry Shrubland	79°52′48.30″E	8°16′23.10″N
Rajanganaya	Tropical Dry Shrubland	80°11′47.20″E	8° 9′56.59″N
Galkadawala	Tropical Dry Forest	80°41′33.07″E	8° 1′52.87″N
Maduru oya	Tropical Dry Forest	81°13′17.15″E	7°38′10.15″N
Kuchchaweli	Tropical Dry Shrubland	81° 5′50.67″E	8°48′53.94″N
Henanigala	Tropical Dry Forest	81° 5′31.11″E	7°35′3.61″N
Wanduragala	Tropical Heavily Degraded Former Forest	80°56′40.58″E	7° 5′14.89″N
Dabaranduwa	Tropical Dry Shrubland	81°11′19.23″E	7°38′45.26″N
Udawalawa	Tropical Dry Shrubland	80°54′7.92″E	6°25′47.79″N
Udawalawa	Tropical Dry Shrubland	80°53′55.77″E	6°28′28.88″N
Balangoda	Tropical Moist Shrubland	80°42′17.31″E	6°40′0.70″N
Alahara	Tropical Dry Forest	80°47′36.47″E	7°44′3.78″N
Kimbissa	Tropical Dry Shrubland	80°43′37.15″E	7°56′14.37″N
Habarana	Tropical Dry Forest	80°45′6.94″E	8° 1′55.97″N
Kaudulla	Tropical Dry Forest	80°51′15.08″E	8° 6′9.76″N
Diyabeduma	Tropical Dry Shrubland	80°52′15.53″E	7°55′58.31″N
Angamadilla	Tropical Dry Forest	80°55′14.79″E	7°55′31.60″N
Habarana	Tropical Dry Forest	80°44′55.25″E	8° 2′12.71″N
Ranawarapitiya	Tropical Dry Shrubland	80°53′51.89″E	8° 7′22.17″N
Ambalanthota	Tropical Heavily Degraded Former Forest	81°01′52.4″E	6°06′57.2″N
Mirijjawila	Tropical Dry Forest	81°05′00.3″E	6°07′04.3″N
Walsapugala	Tropical Dry Forest	81°05′32.7″E	6°14′48.8″N
Vilmanna	Tropical Dry Forest	81°12′44.5″E	6°11′58.9″N
Katharagama	Tropical Dry Forest	81°20′47.8″E	6°25′00.4″N
Thanamalvila	Tropical Dry Forest	81°08′14.3″E	6°26′52.9″N
Wiravila	Tropical Dry Forest	81°13′42.5″E	6°15′38.9″N
Kimbul ara	Tropical Dry Forest	81°08′11.3″E	6°25′16.6″N
Wellawaya	Tropical Dry Grassland	81°06′24.4″E	6°44′27.4″N
Lunugamwehera	Tropical Dry Forest	81°16′07.2″E	6°30′43.3″N
Katharagama	Tropical Dry Forest	81°19′08.9″E	6°24′58.7″N
Dambagalla	Tropical Dry Grassland	81°22′53.3″E	6°59′31.8″N
Bibila	Tropical Dry Grassland	81°21′12.4″E	6°51′38.9″N
Buddangala	Tropical Dry Forest	81°12′40.8″E	6°12′00.7″N
Wanathawilluwa	Tropical Dry Forest	79°51′24.9″E	8°11′14.1″N
Galagedara	Tropical Moist Lowland Forest	80°32′05.9″E	7°22′30.7″N
Medadumbara	Tropical Moist Montane Forest	80°50′29.7″E	7°19′45.2″N
Samanpura	Tropical Heavily Degraded Former Forest	80°23′27.0″E	6°32′03.8″N
Kamburupitiya	Plantations	80°33′53.9″E	6°05′37.6″N
Hakmana	Plantations	80°39′44.1″E	6°05′51.7″N
Deyiyandara	Tropical Moist Lowland Forest	80°36′26.1″E	6°09′11.9″N
Bandagiriya	Tropical Dry Forest	81°10′04.7″E	6°16′15.9″N
Thammannawa	Tropical Heavily Degraded Former Forest	80°35′30.9″E	8°22′29.0″N
Suriyawewa	Tropical Dry Forest	81°01′31.4″E	6°17′56.1″N
Gannoruwa	Plantations	80°35′19.4″E	7°17′28.4″N
Ambalanthota	Plantations	81°01′59.8″E	6°08′25.7″N
Katagamuwa	Tropical Dry Forest	81°22′30.3″E	6°24′04.4″N
Galge	Tropical Dry Grassland	81°17′46.4″E	6°32′04.0″N
Nimalawa	Tropical Dry Grassland	80°25′41.2″E	6°04′03.4″N
Bundala	Tropical Heavily Degraded Former Forest	81°12′38.9″E	6°11′59.1″N
Yala	Tropical Dry Forest	81°32′14.8″E	6°26′12.3″N
Elagolla	Tropical Moist Montane Forest	80°47′08.4″E	7°20′45.0″N
Ampara	Tropical Dry Grassland	81°35′32.2″E	7°18′04.8″N
Batticaloa	Tropical Dry Grassland	81°35′26.2″E	7°42′13.8″N
Minipe	Tropical Dry Grassland	81°00′12.9″E	7°13′51.4″N
Kaballabokka	Tropical Heavily Degraded Former Forest	81°28′13.0″E	7°13′41.6″N
Palabaddala	Tropical Moist Lowland Forest	80°27′08.7″E	6°47′48.2″N
Kuruwita	Tropical Moist Lowland Forest	80°22′58.9″E	6°47′29.6″N
Veheragala	Tropical Dry Shrubland	80°19′41.2″E	7°42′51.1″N
Athimale	Tropical Dry Grassland	81°02′45.3″E	6°52′07.9″N
Kotiyagala	Tropical Dry Grassland	81°30′52.7″E	6°46′57.7″N
Thenagallanda	Tropical Dry Grassland	81°23′31.7″E	6°48′03.8″N
Marawa	Tropical Heavily Degraded Former Forest	80°27′48.5″E	7°15′14.7″N
Kahambana	Tropical Dry Grassland	81°22′49.5″E	6°46′02.5″N
Okkampitiya	Tropical Moist Shrubland	81°18′26.5″E	6°45′07.1″N
Maligawila	Tropical Moist Lowland Forest	81°21′05.0″E	6°43′44.4″N
Lahugala	Tropical Dry Shrubland	81°42′35.0″E	6°52′42.5″N
Kumana	Tropical Dry Forest	81°41′40.0″E	6°32′14.0″N
Nilgala	Tropical Dry Forest	81°15′44.4″E	6°42′38.3″N
Mullegama	Tropical Heavily Degraded Former Forest	80°00′39.2″E	6°53′01.9″N
Baduluwela	Tropical Dry Grassland	81°32′21.8″E	7°09′51.4″N
Bibila	Tropical Dry Grassland	81°21′23.3″E	6°51′50.3″N
Iginiyagala	Tropical Dry Grassland	81°32′26.9″E	7°13′31.2″N
Galoya	Tropical Dry Shrubland	81°28′17.8″E	7°13′27.6″N
Akkarapathtuwa	Tropical Dry Shrubland	81°50′43.8″E	7°14′10.4″N
Panama	Tropical Dry Grassland	81°48′14.6″E	6°45′52.6″N
Haldummulla	Tropical Moist Montane Forest	80°53′05.1″E	6°45′32.7″N
Seelathanna	Tropical Moist Montane Forest	80°53′25.3″E	6°46′01.4″N
Beragala	Tropical Moist Montane Forest	80°54′50.6″E	6°45′56.1″N
Mayillaththawa	Tropical Dry Forest	80°45′48.4″E	7°56′33.8″N
Makulugolla	Tropical Dry Grassland	81°00′37.9″E	7°24′56.6″N
Mahawewa	Tropical Dry Shrubland	79°48′43.3″E	7°27′30.9″N
Norwood	Tropical Moist Montane Forest	80°37′11.5″E	6°50′30.3″N
Narigama	Tropical Moist Shrubland	80°07′04.9″E	6°07′40.9″N
Waddagala	Tropical Moist Lowland Forest	80°30′01.5″E	6°23′26.8″N
Ruunakanda	Tropical Moist Lowland Forest	80°19′02.2″E	6°27′26.4″N
Baduraliya	Tropical Moist Lowland Forest	80°14′12.1″E	6°31′35.3″N
Mathugama	Tropical Moist Shrubland	80°07′12.2″E	6°32′29.4″N
Payagala	Tropical Moist Shrubland	79°59′46.1″E	6°31′10.1″N
Yatadola	Tropical Moist Shrubland	80°04′59.1″E	6°31′31.2″N
Kalupahana	Tropical Moist Shrubland	80°09′45.9″E	6°28′58.0″N
Somawathi	Tropical Dry Grassland	81°10′23.6″E	8°07′25.5″N
Mihinthalaya	Tropical Dry Forest	80°29′34.4″E	8°22′32.0″N
Mullaitivu	Tropical Dry Shrubland	80°49′00.2″E	9°15′51.6″N
Thanthirimale	Tropical Dry Grassland	80°15′21.3″E	8°34′09.2″N
Mahawilachchiya	Tropical Dry Grassland	80°10′46.7″E	8°26′22.7″N
Eluwankulama	Tropical Dry Grassland	79°53′34.8″E	8°17′06.4″N
Kalaoya	Tropical Dry Grassland	80°06′21.7″E	8°12′17.6″N
Kukulkatuwa	Tropical Dry Shrubland	80°09′01.8″E	8°21′58.6″N
Dambana	Tropical Dry Grassland	81°08′34.7″E	7°24′42.0″N
Kotuaththawala	Tropical Dry Shrubland	80°07′15.6″E	7°48′04.2″N
Ibbagamuwa	Tropical Dry Shrubland	80°26′47.6″E	7°33′17.4″N
Maimbul kanada	Tropical Moist Lowland Forest	80°07′09.6″E	7°08′15.3″N
Wasgamuwa	Tropical Dry Shrubland	80°55′11.8″E	7°45′14.4″N
Rathkinda	Tropical Dry Shrubland	81°05′08.0″E	7°31′60.0″N
Manampitiya	Tropical Dry Shrubland	81°07′48.5″E	7°54′30.2″N
Weheragala	Rural gardens	81°15′54.5″E	6°32′56.0″N
Ududumbara	Plantations	80°53′10.2″E	7°15′50.6″N
Alla	Tropical Moist Montane Forest	81°02′57.0″E	6°51′30.2″N
Wallawaya	Tropical Dry Grassland	81°06′18.6″E	6°44′36.2″N
Migalawa	Tropical Dry Grassland	80°23′17.6″E	8°01′43.6″N
Karandugala	Tropical Dry Grassland	81°19′57.4″E	7°16′04.2″N
Pitakumbura	Tropical Dry Grassland	81°17′28.1″E	7°11′01.1″N
Buddangala	Tropical Dry Shrubland	81°41′55.8″E	7°19′06.8″N
Thelijjawila	Tropical Moist Shrubland	80°29′32.4″E	6°01′33.2″N
Kanneliya	Tropical Moist Lowland Forest	80°20′05.4″E	6°14′56.4″N
Dekwella	Plantations	80°41′23.4″E	5°58′36.2″N
Walihinda	Tropical Moist Lowland Forest	79°59′45.4″E	6°55′21.9″N
Yagirala	Tropical Moist Lowland Forest	80°09′44.9″E	6°22′20.4″N
Aviththawa	Tropical Moist Lowland Forest	80°08′49.6″E	6°22′12.0″N
Elpitiya	Tropical Moist Shrubland	80°09′48.0″E	6°18′07.5″N
Kalupahana	Tropical Moist Lowland Forest	80°09′51.1″E	6°28′46.7″N
Horapawita	Tropical Moist Lowland Forest	80°35′20.7″E	6°06′31.9″N
Kumbalgoda	Tropical Moist Shrubland	80°41′02.9″E	6°03′52.2″N
Mulatiyana	Tropical Moist Lowland Forest	80°35′21.2″E	6°10′19.2″N
Maththala	Tropical Dry Grassland	81°06′40.4″E	6°17′52.9″N
Kahatagas degiliya	Tropical Dry Grassland	80°41′54.7″E	8°23′57.5″N
Andara wewa	Tropical Dry Shrubland	81°04′46.6″E	6°19′04.8″N
Phimbiyagollewa	Tropical Dry Shrubland	80°37′05.4″E	8°30′45.8″N
Karawita	Tropical Moist Lowland Forest	80°25′17.1″E	6°35′10.5″N
Hangamuwa	Tropical Moist Shrubland	80°24′16.2″E	6°38′30.5″N
Henegama	Tropical Moist Lowland Forest	80°27′12.8″E	6°03′20.9″N
Pahuranwila	Tropical Moist Shrubland	80°28′39.0″E	6°04′19.9″N
Kirinda	Tropical Dry Grassland	81°20′31.2″E	6°14′04.8″N
Galkaduwa	Tropical Dry Grassland	81°22′52.7″E	6°19′50.3″N
Rikillagaskada	Tropical Moist Montane Forest	80°48′11.0″E	7°09′13.2″N
Poramadulla	Tropical Moist Lowland Forest	80°46′46.3″E	7°08′00.5″N
Kegalle	Tropical Moist Lowland Forest	80°18′53.9″E	7°15′26.3″N
Chenkaladi	Tropical Dry Grassland	81°33′50.4″E	7°46′56.8″N
Mangalagama	Tropical Dry Grassland	81°30′41.3″E	7°30′23.3″N
Bakkiela	Tropical Moist Montane Forest	81°36′00.4″E	7°29′08.5″N
Erathna	Tropical Moist Montane Forest	80°24′14.3″E	6°49′55.1″N
Heerallugama	Tropical Dry Forest	80°38′00.1″E	8°37′04.1″N
Thanthirimale	Tropical Dry Forest	80°18′56.4″E	8°34′01.7″N
Okkampitiya	Tropical Dry Shrubland	81°20′34.8″E	6°46′07.8″N
Hulandawa	Tropical Dry Shrubland	81°20′12.1″E	6°50′47.0″N
Wattegama	Tropical Dry Shrubland	81°28′41.0″E	6°46′59.9″N
Bingiriya	Tropical Dry Shrubland	79°56′37.4″E	7°36′52.0″N
Bangadeniya	Tropical Dry Shrubland	79°50′13.0″E	7°37′58.8″N
Boraluwewa	Tropical Dry Shrubland	80°03′21.0″E	7°41′38.3″N
Welpothuwewa	Tropical Dry Shrubland	80°01′33.2″E	7°42′20.6″N
Ambanpola	Tropical Dry Shrubland	80°12′23.3″E	7°56′40.5″N
Dodangoda	Plantations	80°01′59.8″E	6°33′07.0″N
Mahaoya	Tropical Dry Forest	81°22′30.9″E	7°39′04.4″N
Dimbulagala	Tropical Dry Forest	81°12′20.1″E	7°46′33.6″N
Sungavila	Tropical Dry Forest	81°06′47.3″E	8°05′01.6″N
Kanthale	Tropical Dry Forest	80°59′55.2″E	8°24′12.8″N
Welioya	Tropical Dry Forest	80°45′11.9″E	8°57′31.5″N
Nelumkulama	Tropical Dry Forest	80°05′43.0″E	6°58′12.7″N
Lankapura	Tropical Dry Forest	81°06′46.8″E	8°00′24.9″N
Medirigiriya	Tropical Dry Forest	81°03′53.0″E	8°10′45.8″N
Wathurugama	Plantations	80°06′34.3″E	7°02′43.7″N

**Table 2:** Data received from DWC offices on reported the crimes involving pangolins includes date of crime, approximate GPS coordinates of areas where crimes occurred, type of crimes and the fines charged.

DWC office	Date	Longitude	Latitude	Type of Crime	Fine (LKR)
Ritigala Strict Nature Reserve	13-Apr-2017	80°37′40.50″E	8° 5′15.40″N	Possession of flesh	30000.00
Kakirawa Range	16-Feb-2016	80°31′57.57″E	8° 4′22.72″N	Possession of flesh	On going
Vavuniya Range	23-Nov-2010	80°58′53.30″E	8°52′29.40″N	Possession of flesh	30000.00
Vavuniya Range	8-Sep-2012	80°12′15.28″E	8°51′22.90″N	Possession of cooked flesh	10 days community service
Horowupothana Elephant Detention Center	13-Aug-2017	80°50′6.04″E	8°33′33.48″N	Possession of flesh	30000.00
Anuradhapura Regional	15-Nov-2013	80°21′4.88″E	8°18′28.35″N	Killing of pangolin within protected area	240000.00
Anuradhapura Regional	13-Jan-2015	80°30′16.43″E	8°21′8.75″N	Possession of flesh and explosive	80000.00
Anuradhapura Regional	15-Feb-2015	80°31′15.23″E	8°22′9.35″N	Possession of flesh	75000.00
Anuradhapura Regional	14-Jan-2015	80°35′24.90″E	8°22′50.89″N	Possession of cooked flesh	75000.00
Wilpattu National Park	3-Jan-2011	79°52′48.30″E	8°16′23.10″N	Possession of flesh	30000.00
Wilpattu National Park	10-May-2008	80°11′47.20″E	8° 9′56.59″N	Possession of scales	20000.00
Wilpattu National Park	12-May-2006	80°16′34.22″E	8° 9′34.56″N	Possession of flesh	30000.00
Wilpattu National Park	6-Jan-2010	80°41′33.07″E	8° 1′52.87″N	Possession of flesh and explosive	Not mentioned
Ulhitiya Range	18-Sep-2002	81°13′17.15″E	7°38′10.15″N	Possession of flesh, unauthorized entry to the National Park and possession of weapons	Two years imprisonment and paused imprisonment for five years
Ulhitiya Range	31-Dec-2010	81° 8′45.02″E	7°31′13.91″N	Possession of flesh	Paused imprisonment for Five years
Pigeon Island National Park	5-Aug-2017	81° 5′50.67″E	8°48′53.94″N	Killing of pangolin	30000.00
Maduru oya National Park	28-Mar-2004	81° 5′31.11″E	7°35′3.61″N	Possession of flesh and exhibit for sale	25000.00
Maduru oya National Park	18-Sep-2002	80°56′40.58″E	7° 5′14.89″N	Possession of flesh and weapons	Not mentioned
Maduru oya National Park	31-Dec-2014	81°11′14.33″E	7°38′52.47″N	Possession of flesh and weapons	70000.00
Maduru oya National Park	29-Jun-2017	81°11′14.33″E	7°38′52.47″N	Possession of flesh	On going
Maduru oya National Park	6-May-2017	81° 5′31.11″E	7°35′3.61″N	Possession of flesh	30000.00
Maduru oya National Park	29-Aug-2007	81°11′19.23″E	7°38′45.26″N	Attempt of capture within protected area	30000.00
Udawalawa National Park	3-Aug-2016	80°54′7.92″E	6°25′47.79″N	Possession of flesh and exhibit for sale	60000.00
Udawalawa National Park	23-Dec-2000	80°53′53.76″E	6°28′24.85″N	Killing of pangolin	Not mentioned
Udawalawa National Park	25-Apr-2001	80°53′55.77″E	6°28′28.88″N	Possession of flesh	Not mentioned
Udawalawa National Park	3-Aug-2003	80°53′55.77″E	6°28′28.88″N	Possession of flesh	Not mentioned
Samanala Range	14-Oct-2014	80°42′17.31″E	6°40′0.70″N	Possession of live pangolin	35000.00
Alahara Range	21-Apr-2008	80°47′36.47″E	7°44′3.78″N	Possession of live pangolin	15000.00
Segiriya Range	13-Feb-2010	80°43′37.15″E	7°56′14.37″N	Possession of flesh	20000.00
Minneriya National Park	14-Jan-2002	80°45′6.94″E	8° 1′55.97″N	Possession of flesh	Not mentioned
Minneriya National Park	2-Nov-2002	80°51′15.08″E	8° 6′9.76″N	Possession of flesh and weapons	20000.00
Minneriya National Park	13-Apr-2005	80°54′23.01″E	8° 2′19.22″N	Killing of pangolin	Not mentioned
Minneriya National Park	20-Jun-2013	80°52′15.53″E	7°55′58.31″N	Possession of flesh	30000.00
Angamadilla National Park	20-Aug-2015	80°55′14.79″E	7°55′31.60″N	Killing of pangolin	Not mentioned
Kaudulla National Park	18-Feb-2008	80°44′55.25″E	8° 2′12.71″N	Possession of flesh and exhibit for sale	Not mentioned
Kaudulla National Park	14-Jan-2017	80°53′51.89″E	8° 7′22.17″N	Possession of flesh	100000.00
Hambanthota Range	29-Jun-2011	81°01′52.4″E	6°06′57.2″N	Possession of flesh	80000.00
Hambanthota Range	27-May-2013	81°05′00.3″E	6°07′04.3″N	Possession of flesh	30000.00
Hambanthota Range	15-May-2016	81°05′32.7″E	6°14′48.8″N	Electrocution of pangolin due to unsafely located private electric fence	30000.00
Bundala National Park	24-Jun-2014	81°12′44.5″E	6°11′58.9″N	Possession of flesh	80000.00
Uva Province Regional	7-Dec-2007	81°20′47.8″E	6°25′00.4″N	Possession of flesh	15000.00
Lunugamvehera Range	13-Oct-2011	81°08′14.3″E	6°26′52.9″N	Possession of flesh	15000.00
Lunugamvehera Range	8-Nov-2001	81°13′42.5″E	6°15′38.9″N	Possession of live pangolin	Not mentioned
Lunugamvehera Range	5-Mar-2005	81°08′11.3″E	6°25′16.6″N	Possession of flesh	12500.00
Lunugamvehera Range	22-Jun-2006	81°06′24.4″E	6°44′27.4″N	Possession of flesh	15000.00
Lunugamvehera Range	27-Oct-2013	81°16′07.2″E	6°30′43.3″N	Possession of flesh	90000.00
Galge Range	21-Sep-2007	81°19′08.9″E	6°24′58.7″N	Possession of scales	15000.00
Gal oya National Park	15-Jan-2017	81°22′53.3″E	6°59′31.8″N	Possession of flesh	On going
Gal oya National Park	21-Feb-2017	81°21′12.4″E	6°51′38.9″N	Possession of skin	On going
Ampara Range	14-Feb-2018	81°12′40.8″E	6°12′00.7″N	Possession of live pangolin	50000.00
Northwest Regional	29-Jul-2107	79°51′24.9″E	8°11′14.1″N	Possession of cooked flesh	30000.00
Randenigala Range	10-Jul-2016	80°32′05.9″E	7°22′30.7″N	Possession of flesh and exhibit for sale	80000.00
Central Regional	14-Oct-2014	80°45′54.3″E	7°19′19.8″N	Possession of flesh	35000.00
Waddagala Range	13-Feb-2013	80°23′27.0″E	6°32′03.8″N	Possession of flesh	50000.00

**Table 3:** Data on the areas where rescue activities of Indian Pangolins were carried out in Sri Lanka; approximate GPS coordinates of those areas; health conditions at the rescue and post rescue.

Area	Longitude	Latitude	Health condition at rescue	Health conditions at post rescue
Kanakapuram	80°23′14.07″E	9°23′0.27″N	Healthy	Released to the same range
Kilinochchi	80°22′37.20″E	9°22′49.04″N	Healthy	Released to different range
Trincomalee	81°12′54.76″E	8°35′14.51″N	Healthy	Released to the same range
Kahatagas wewa	80°53′25.83″E	8°26′42.41″N	Healthy	Released to different range
Puliyankulama	80°34′44.27″E	8°28′38.25″N	Healthy	Released to the same range
Kakerawa	80°35′37.80″E	8° 2′31.57″N	Healthy	Released to different range
Palugas wewa	80°41′17.42″E	8° 3′37.59″N	Diseased	Died while receiving treatment
Mullaitivu	80°48′47.3″E	9°15′50.9″N	Diseased	Died while receiving treatment
Wilpattu	80° 2′51.50″E	8°27′22.94″N	Diseased	Died while receiving treatment
Hunuvila gama	80°10′11.30″E	8°19′2.14″N	Diseased	Died while receiving treatment
Nikaweratiya	80° 7′54.09″E	7°44′46.99″N	Healthy	Released to the same range
Maduru oya	81°11′9.67″E	7°39′24.87″N	Healthy	Released to the same range
Panamure	80°46′6.78″E	6°20′37.77″N	Healthy	Released to different range
Kalamatiya	80°56′14.50″E	6° 5′31.87″N	Injured	Died while receiving treatment
Udawalawa	80°49′26.28″E	6°25′6.61″N	Injured	Died while receiving treatment
Kolambage ara	80°46′49.17″E	6°25′6.08″N	Healthy	Released to different range
Dikwella	80°41′42.38″E	5°58′18.04″N	Diseased	Died while receiving treatment
Kahawatta	80°34′27.83″E	6°34′57.18″N	Injured	Died while receiving treatment
Lellopitiya	80°28′55.09″E	6°40′0.29″N	Injured	Died while receiving treatment
Udawalawa	80°48′16.65″E	6°28′20.57″N	Healthy	Released to different range
Kalthota	80°52′14.55″E	6°39′32.84″N	Healthy	Released to different range
Balangoda	80°42′17.31″E	6°40′0.70″N	Diseased	Died while receiving treatment
Dahaiyagala	80°34′13.00″E	7°31′45.99″N	Diseased	Died while receiving treatment
Kalthota	80°52′15.55″E	6°39′33.84″N	Injured	Died while receiving treatment
Palabaddala	80°27′35.45″E	6°47′42.06″N	Healthy	Released to different range
Balangoda	80°40′1.45″E	6°38′59.19″N	Healthy	Released to the same range
Polpithigama	80°24′42.47″E	7°48′45.16″N	Healthy	Released to different range
Kurunagala	80°21′43.68″E	7°32′27.05″N	Diseased	Died while receiving treatment
Malkaduwawa	80°20′9.08″E	7°28′7.68″N	Diseased	Died while receiving treatment
Giriulla	80° 7′23.95″E	7°19′54.37″N	Healthy	Released to different range
Rajanganaya	80°11′47.20″E	8° 9′56.59″N	Diseased	Died while receiving treatment
Balangoda	80°41′1.43″E	6°38′56.19″N	Healthy	Released to different range
Nikawaratiya	80° 7′54.09″E	7°44′46.99″N	Healthy	Released to different range
Nikawaratiya	80° 7′54.09″E	7°44′46.99″N	Healthy	Released to different range
Polpithigama	80°24′42.65″E	7°48′50.64″N	Diseased	Died while receiving treatment
Polpithigama	80°26′54.01″E	7°51′36.36″N	Healthy	Released to different range
Kobeyigane	80° 7′33.99″E	7°39′18.49″N	Healthy	Released to different range
Yapahuwa	80°17′55.37″E	7°49′39.56″N	Diseased	Died while receiving treatment
Kurunagala	80°21′59.41″E	7°30′58.42″N	Injured	Died while receiving treatment
Thiththawella	80°20′50.78″E	7°30′59.06″N	Healthy	Released to different range
Sigiriya	80°45′36.58″E	7°57′18.18″N	Healthy	Released to different range
Katukaliyawa	80°35′18.31″E	8°21′8.74″N	Healthy	Released to different range
Sigiriya	80°45′48.04″E	7°57′27.35″N	Healthy	Released to the same range
Bandimaawa	80°47′18.33″E	8° 0′19.12″N	Healthy	Released to the same range
Diyabeduma	80°53′59.42″E	7°56′38.39″N	Healthy	Released to the same range
Trincomalee	81°10′35.75″E	8°33′19.53″N	Injured	Died while receiving treatment
Minneriya	80°54′44.43″E	8° 3′9.99″N	Healthy	Released to the same range
Herathgama	80°12′15.18″E	7°39′50.69″N	Diseased	Died while receiving treatment
Pandulagama	80°21′16.45″E	8°19′40.66″N	Injured	Died while receiving treatment
Habarana	80°44′28.70″E	8° 2′12.09″N	Healthy	Released to the same range
Herathgama	80°12′12.16″E	7°39′51.67″N	Healthy	Released to different range
Minneriya	80°54′40.01″E	8° 2′48.55″N	Healthy	Released to the same range
Deyiyandara	80°36′07.9″E	6°09′16.3″N	Diseased	Died while receiving treatment
Denagama	80°39′38.9″E	6°06′29.4″N	Diseased	Died while receiving treatment
Tissa	81°14′28.0″E	6°17′52.8″N	Healthy	Released to different range
Yatiyana	80°35′54.5″E	6°01′29.6″N	Healthy	Released to the same range
Tissa	81°16′28.0″E	6°18′52.7″N	Healthy	Released to the same range
Lunugamvehera	81°12′04.3″E	6°21′10.5″N	Diseased	Died while receiving treatment
Ranawaranawa	81°08′55.2″E	6°24′24.6″N	Healthy	Released to the same range
Wallawaya	81°05′49.2″E	6°44′02.8″N	Healthy	Released to the same range
Okkampitiya	81°18′37.4″E	6°45′27.9″N	Diseased	Died while receiving treatment
Mullegama	80°00′39.4″E	6°53′04.2″N	Diseased	Died while receiving treatment
Paranigama	81°05′49.1″E	8°03′51.4″N	Healthy	Released to the same range
Kethsirigama	81°39′36.2″E	7°12′28.1″N	Healthy	Released to the same range
Nawaththadu	79°50′34.7″E	8°10′00.7″N	Healthy	Released to the same range
Norochcholai	79°42′54.5″E	8°02′30.4″N	Healthy	Released to the same range
Ilanthadiya	79°42′52.6″E	8°04′03.7″N	Healthy	Released to the same range
Ilanthadiya	79°43′36.9″E	8°10′39.0″N	Healthy	Released to the same range
Ekkala	79°51′01.4″E	8°08′47.9″N	Healthy	Released to the same range
Baduraliya	80°14′20.4″E	6°31′02.0″N	Healthy	Released to the same range
Badugama	80°07′17.1″E	6°32′25.7″N	Healthy	Released to the same range
Miriswaththa	80°08′43.1″E	6°57′41.2″N	Injured	Died while receiving treatment
Wanduramba	80°15′48.5″E	6°07′58.4″N	Healthy	Released to the same range
Unawatuna	80°15′42.6″E	6°00′33.2″N	Healthy	Released to the same range
Wanduramba	80°14′44.9″E	6°08′29.7″N	Healthy	Released to the same range
Baddegama	80°11′31.6″E	6°10′37.5″N	Healthy	Released to the same range
Karandeniya	80°06′10.2″E	6°16′26.1″N	Healthy	Released to the same range
Poddala	80°12′46.5″E	6°07′27.7″N	Healthy	Released to the same range
Yakkalamulla	80°21′09.6″E	6°05′30.4″N	Healthy	Released to the same range
Alpitiya	80°09′13.6″E	6°17′17.8″N	Healthy	Released to the same range
Udugama	80°21′02.8″E	6°14′09.1″N	Healthy	Released to the same range
Imaduwa	80°23′10.0″E	6°02′05.8″N	Healthy	Released to the same range
Wattakapola	80°39′12.3″E	7°21′06.0″N	Injured	Died while receiving treatment
Manikhinna	80°42′33.0″E	7°18′56.4″N	Healthy	Released to the same range
Wirambugedara	80°14′22.7″E	7°28′35.4″N	Injured	Died while receiving treatment
Katupotha	80°10′30.0″E	7°32′50.9″N	Healthy	Released to the same range
Horambawa	80°11′11.1″E	7°28′51.4″N	Healthy	Released to the same range
Hettipola	80°04′46.0″E	7°35′43.4″N	Injured	Died while receiving treatment
Narammala	80°14′50.6″E	7°25′05.2″N	Healthy	Released to the same range
Hettipola	80°04′45.0″E	7°35′49.9″N	Healthy	Released to the same range
Madalagama	80°30′05.3″E	6°33′53.8″N	Healthy	Released to the same range
Wariyapola	80°24′17.4″E	7°37′15.3″N	Healthy	Released to different range
Kuliyapitiya	80°03′30.1″E	7°28′32.2″N	Healthy	Released to the same range
Pallegama	80°03′25.7″E	7°17′44.6″N	Healthy	Released to the same range
Thalageya	80°17′34.3″E	7°28′03.3″N	Healthy	Released to the same range
Hulogedara	80°11′19.4″E	7°47′45.3″N	Healthy	Released to the same range
Nillegama	80°09′50.5″E	7°45′57.3″N	Healthy	Released to the same range
Kobeyigane	80°08′00.8″E	7°39′22.7″N	Healthy	Released to the same range
Galaha	80°35′43.0″E	7°16′03.1″N	Healthy	Released to the same range
Galway's Land	80°46′37.2″E	6°58′01.1″N	Healthy	Released to different range
Hingurukaduwa	81°08′25.3″E	6°49′16.7″N	Healthy	Released to the same range
Bandarawela	80°59′30.2″E	6°49′07.8″N	Healthy	Released to the same range
Diyathalawa	80°57′59.2″E	6°47′57.7″N	Injured	Died while receiving treatment
Thelijjawila	80°29′35.9″E	6°01′43.1″N	Injured	Died while receiving treatment
Waligama	80°29′25.5″E	6°01′38.3″N	Healthy	Released to the same range
Walihinda	80°28′02.9″E	5°58′54.4″N	Injured	Died while receiving treatment
Yakkalamulla	80°21′48.4″E	6°06′36.9″N	Injured	Died while receiving treatment
Udugama	80°20′40.3″E	6°13′53.5″N	Injured	Died while receiving treatment
Udawalawa	80°48′11.5″E	6°26′45.2″N	Diseased	Died while receiving treatment
Iginiyagala	81°33′05.8″E	7°12′04.4″N	Diseased	Died while receiving treatment
Uhana	81°38′09.8″E	7°22′25.9″N	Healthy	Released to the same range
Galapita gala	81°39′30.7″E	7°28′02.5″N	Healthy	Released to the same range
Maduru oya	81°12′58.1″E	7°38′57.0″N	Healthy	Released to the same range
Ampara	81°40′04.2″E	7°13′59.7″N	Injured	Died while receiving treatment

### 1.1 Data on the distribution

Data on the distribution (Table 1) include location information gathered by direct and indirect field observations of the research team and location information extracted from rescue and wildlife crime records of the Department of Wildlife Conservation, Sri Lanka.

### 1.2 Data on crime records

Data on crime records involving Indian pangolins (Table 2) include information extracted from registries of DWC offices pertaining to the period January 2000 to December 2018.

### 1.3 Data on rescue records

Data on rescue records of Indian pangolins (Table 3) include information extracted from registries of DWC offices, animal rescue centers and Pinnawala National Zoological Garden, pertaining to the period January 2000 to December 2018.

## 2 Experimental design, materials, and methods

Collection of ecological data on nocturnal elusive mammals such as pangolin is difficult due to lower number of records per significant time period [2]. Thus indirect methods such as camera trap surveys, sign surveys and community surveys are often used by researchers [[3], [4], [5]]. Field data gathered by the authors during ecological surveys to determine the abundance [6,7], and habitat utilization of Indian pangolins [5] in the South-west lowland forest and associated landscapes in Sri Lanka (Yagirala: 6°21′ to 6°26′ N and 80°08′ to 80°11′ E), and two other study locations (Wilpattu: °13′ to 8°40′N and 79°50′ to 80°10′E and Yala: 6°18′ to 6°42′N and 81°24–81°43′E) from August 2013 to December 2018 were used as primary data on pangolin recordings. We collected data on the distribution, rescue and wildlife crimes involving Indian pangolins from January 2000 to December 2018 to update the distribution and conservation status of *Manis crassicaudata* in Sri Lanka. Indian pangolin is the only pangolin species in Sri Lanka [8] and it has a wide distribution from coastal areas to central hills [7]. The data on the distribution, habitats, crimes and rescue activities involving this species are dispersed throughout the country. Hence, an island-wide data collection method from multiple sources was employed for a comprehensive assessment. Owing to the elusive nature of the animal, locals are less aware about the Indian pangolin [9]. Thus, relying only on community surveys to gather data on distribution can generate biased information. Therefore, to obtain more accurate information, the data was collected from the registries of government and nongovernmental stake holder institutions committed to the conservation of wildlife. The data records from the mass media such as newspapers, web, television and radio were also included to the respective data sets after verifying with responsible authorities. The data on crime records in Table 2 was collected from the offense records maintained by the beet/range/national Park/regional offices of the DWC. The records on rescue activities in Table 3 was collected from the rescue records maintained by the aforesaid offices of DWC and animal health care centers managed by both DWC and nongovernmental organizations. Further, data from the rescue registry of Pinnawala National Zoological Garden, Sri Lanka, was also added to data on rescue activities because it is directly involved in the rescue of pangolins from its surrounding areas. The data on the distribution in Table 1 was produced by combining the data of both rescue and crime activities as well as the verified data collected from field and mass media records involving pangolins.
